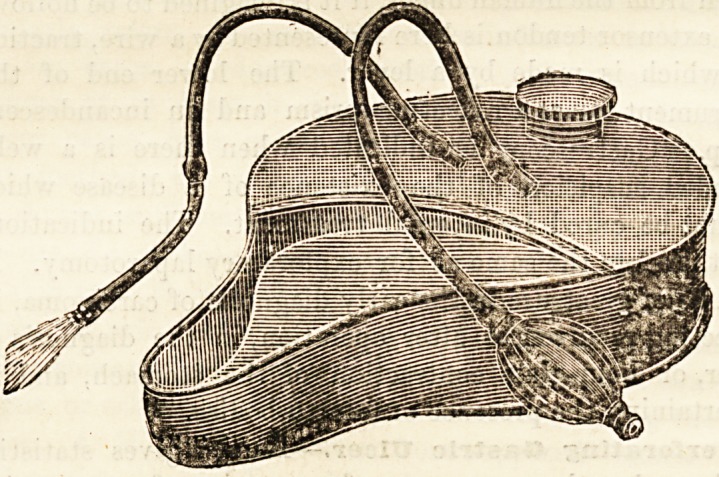# New Appliances and Things Medical

**Published:** 1901-01-26

**Authors:** 


### NEW APPLIANCES AND THINGS MEDICAL.

[We shall be glad to receive, at our Office, 28 & 29 Southampton Street, Strand, London, W.C., from the manufacturers, specimens of all new preparations and appliances which may be brought out from time to time.]

### STERILISED ABSORBENT RIBBON GAUZE AND BATTIST.

(GALEN MANUFACTURING CO., LTD., LADYWELL, LONDON, S.E.)

THE ribbon gauze has been introduced by the manufacturers at the suggestion of Mr. Stonham, and is intended to be used in place of ordinary strips of the material which are usually cut to the required size by the surgeon or nurse. Such cut strips have necessarily a frayed edge, and consequently often leave small threads or strands of threads in the wound which has been plugged. In deep sinuses or fistulous openings this is a real danger, and may lead to subsequent irritation and interfere with the production of normal granulation tissue and healing of the wound. Milne's ribbon gauze, which can be obtained in several widths, has a firm woven edge, and hence is free from the above objection. It is a well-manufactured article, particularly soft, and if so required by the surgeon can be impregnated with any antiseptic and in any strength. The Battist is a substitute for the various protective oiled silks generally employed to save the bandage or outer dressing from the greasy or moist materials in contact with the wound. It has the advantage of being grease-proof, spirit-proof, and waterproof, and further it can be boiled without injury, and will withstand the action of most antiseptic preparations. A protective dressing with the above advantages which can be procured at the reasonable price which is quoted by the manufacturer should ensure Milne's Battist an extensive use in hospitals and in private practice.

### A COMBINED DOUCHE AND BIDET.

(W. H. BAILEY AND SON, 38 OXFORD STREET, LONDON, W.)

THIS is a device which may in certain cases save a good deal of trouble and many wet beds. The idea is to combine in one piece a bed-pan, a water or lotion reservoir, and a douche. The shape and arrangement will be readily understood from the accompanying diagram. The apparatus is made of tin. The pointed part is the receiver, and its uses are sufficiently explained by its conformation. The wider end is cut off by an air-tight division, and so when the cap is screwed down forms an air-tight reservoir for the water or lotion which is to be used. Leading into this reservoir, however, there are two tubes: one, connected with an india-rubber hand-bellows, opens into the top of the reservoir; the other, connected with the vaginal douche pipe, opens into the bottom of the reservoir, and therefore, of course, under water. All being suitably arranged, and the cap covering the inlet having been screwed down, the hand works the bellows, air is thus driven into the reservoir, and water issues in a stream, gentle or forcible, as may be required. It is a pretty idea. No mess, no trouble, no hanging douches, no dangling pipes. We think, however, it might be worth while to insert a valve, or some other means, for preventing the injection of air.

### ICHTHOFORM.

(CORDES, HERMANNI AND CO., HAMBURG. London representative: GUSTAV HERMANNI, Junr., 20 HIGH HOLBORN, LONDON, W.C.)

THIS new drug consists of the combination of formaldehyde and ichthyol; it is an amorphous powder, of a dark slate colour, odourless, tasteless, and insoluble in water. It may be administered in doses of from 10 to 60 grains with perfect safety. It appears to have most valuable properties as an intestinal antiseptic; indeed, in certain cases of ulceration of the bowels it claims an almost specific action. Its powerful antiseptic action on pathogenic micro-organisms is ascribed to the decomposition of the drug into its constituent parts, with the liberation of formaldehyde in a nascent condition. It apparently exercises no noxious influence on the blood, or on the kidneys or other excretory organs. It is particularly indicated in tuberculous ulceration of the intestine, and in cases of diarrhœa due to microbic infection.

## Figures and Tables

**Figure f1:**